# Music therapy for pain and anxiety in patients after cardiac valve replacement: a randomized controlled clinical trial

**DOI:** 10.1186/s12872-023-03058-5

**Published:** 2023-01-18

**Authors:** Yi Dong, Lin Zhang, Liang-Wan Chen, Zeng-Rong Luo

**Affiliations:** 1 Department of Cardiovascular Surgery and Cardiac Disease Center, Union Hospital, Fujian Medical University, Fuzhou, 350001 People’s Republic of China; 2 Key Laboratory of Cardio-Thoracic Surgery (Fujian Medical University), Fujian Province University, Fuzhou, People’s Republic of China

**Keywords:** Anxiety, Pain, Vital signs, Music therapy, Cardiac valve replacement

## Abstract

**Objective:**

This study aimed to assess how listening to music after cardiac valve replacements affected patients' pain, anxiety, and vital signs.

**Method:**

In Fuzhou, China's Fujian Medical University Union Hospital, the cardiac surgery division conducted a randomized controlled clinical experiment. 86 patients were enrolled, and 43 were assigned randomly to each group (control and experimental group). The standard treatment was given to the control group, while the experimental group was given standard treatment + a 15-min music intervention 3 times. Indicators include pain, anxiety and vital signs (respiratory rate, heart rate, and blood pressure).

**Results:**

In comparison to the control group, the experimental group, over time, demonstrated a statistically substantial decrease in pain, anxiety, systolic blood pressure, heart rate and respiratory rate (all *P* < 0.001), yet, there were no discernible variations (*P* > 0.05) in diastolic blood pressure.

**Conclusions:**

In conclusion, these results provide additional proof for using music therapy to minimize cardiac postoperative pain and anxiety, as well as systolic blood pressure, heart rate and respiratory rate. Moreover, it should be regarded as a supplementary treatment for pain and anxiety after cardiac valve replacement and other medical procedures with comparable postoperative pain.

## Introduction

One of the most prevalent symptoms during rehabilitation from cardiac surgery is pain at rest and when receiving routine care [1]. The most unpleasant clinical experience, pain following thoracotomy, is a significant acute traumatic pain resulting from an incision [2]. Aching, blistering, throbbing, tiring, miserable, or intolerable is common forms that patients express during pain [3]. Ineffective pain treatment can cause serious disruptions in bodily or psychological processes, elicit anxiety, upset sleep, impair hemodynamic stability, complicate the participant's condition, and lengthen the duration of their stay [4]. Despite the increasing effort to reduce pain inflicted while in the hospital, the proportion of cardiac patients who receive insufficient pain management is growing [5].

Patients experience a lot of anxiety while in the hospital because anxiety frequently goes hand in hand with pain [6]. Patients with anxiety may lose hope in their ability to fight off diseases, experience helplessness and pessimism, develop more stress, experience high heart rates, lose appetite and weight, struggle to sleep and digest, feel exhausted, and experience other negative effects that hurt the patient and slow their recovery [7]. Therefore, identifying a suitable method of lowering patient stress requires considerably deeper research [6].

To address the issue of excessive intense pain and the negative side effects of conventional opioid-based treatment, professional organizations have produced guidelines for clinical practice that encourage the application of multifunctional therapeutic strategies that may be opioid limiting. [8]

In clinical practice, the use of music therapy as a non-pharmacological supplement has been very widespread [9]. According to studies, listening to music can promote positive emotions and mood, minimize psychiatric symptoms, relieve pain, lower distress, and reduce anxiety [10].

Even though there has been an increase in the number and quality of music therapy investigations in China in recent years, [11] the quality and quantity of research are limited and insufficient, and conflicting outcomes have been documented. We predict that individuals who receive music therapy would experience less discomfort and anxiety than the control group. This study examined how well music improved patients' post-cardiac surgery pain, anxiety, and vital signs to demonstrate additional confirmation that music therapy is advantageous to patients in China.

## Methods and patients

### Study design

From May 2019 to February 2020, a randomized headphones-controlled single-blind trial of patients undergoing elective heart valve replacements was conducted at Fujian Medical University Union Hospital in Fuzhou (China). This study was registered in the Chinese Clinical Trial Registry (Registration date:10/04/2019; Number: ChiCTR1900022408).

### The experimental group

Music therapy was delivered by binaural headphones to the patients three times 2 h after extubation, the second day after transfer to the general ward, and 1 day before discharge in the morning) by two senior experienced researchers. The subjects underwent the same soft music treatment (15 min) while laying in a recumbent position and wearing binaural headphones after a 30-min rest.

### The control group

The control group received binaural headphones for the same three repetitions without any music and also after a 30-min rest.

### Study participants

Participants in the research underwent elective cardiac valve replacement surgical procedures. Inclusion criteria included being: (1) age ≥ 18 years; (2) eager to participate; (3) conscious; and (4) able to answer questions on pain, sleeplessness, and anxiety. Exclusion criteria included conditions putting the patient at higher risk of adverse outcomes, such as: (1) body mass index (BMI) > 35 kg/m^2^); (2) pulmonary artery pressure > 50 mmHg; (3) right ventricular failure; (4) ejection fraction (EF) of ≤ 35%; and (5) intubation for longer than 24 h. Participants who exhibited vision and hearing deficits or underwent emergency surgeries were excluded.

The reasoning behind the exclusion criteria was supported by several earlier research that revealed individuals with low EFs had significantly worse postsurgical findings than subjects with elevated EFs [12]. Furthermore, the risk of postoperative complications was raised by right ventricular failure [13], intubation > 24 h, BMI > 35 kg/m^2^, [14] and pulmonary artery pressure > 50 mmHg. [15] In order to reduce the impact of increased postoperative complications such as acute kidney injury, low cardiac output syndrome and increased intubation duration on assessment of pain, anxiety and vital signs,

### Ethical approval

The Human Ethical Committee of Fujian Medical University Union Hospital approved the study (Approval Number: 2019KY019, date: 2019-01-31).

Participants were informed of the research protocol, and that enrollment was completely optional. Additionally, there would be no consequences if they decided to stop participating in the study at any point during it. Before commencing the experiment, all subjects submitted their informed consent.

### Sample size

The relevant sample size was calculated using the G*Power 3 software [16] to determine whether music therapy may reduce pain intensity. To detect a mean difference in pain intensity scores of 1.5 points (SD = 2.0) [17] shortly after music treatment, a total sample size of 86 patients was needed, with a two-sided significance level of 0.05, a power of 0.90 in a repeated measure between-factors context, considering a 10% dropout rate. 86 individuals (43 in the intervention group and 43 in the control group) were finally examined for this research.

### Randomization

Individuals were randomly placed into the control or experimental groups using a computer-generated list of randomly generated numbers. The group assignments were hidden from the participants. They were only told which of the two groups they would be placed in and that both groups would receive a treatment that involved wearing binaural headphones.

### Measures

The demographic characteristics regarding the study participants were recorded at the time of admission. The participants' baseline sleep quality was obtained by averaging preoperative three measurements of the RCSQ (Richards-Campbell Sleep Questionnaire) score. The vital signs (respiration rate [RR], heart rate [HR], diastolic blood pressure [DBP], and systolic blood pressure [SBP]) were also obtained via ECG monitor and arterial or cuff manometer by averaging three measured results at four corresponding time points respectively.

### Primary outcome

The preintervention pain level and the pain level after the treatment between the two groups were compared using a VAS (visual analogue scale) [18] for pain quantification. The scale runs from 0 to 10, with 0 representing no pain and 10 being the most severe suffering achievable. A clinically significant variation is defined as a 20% or greater change [19]. Individuals can select an expression ranging from "no hurts" to "hurts the worst" to best reflect their discomfort level. Among the five frequently used pain scales for patients after cardiovascular surgery, a study found that VAS had the highest response rate, the most reliable, and the easiest to comprehend [20].

### Secondary outcome

The STAI (state-trait anxiety inventory) was utilized to determine the secondary outcome: anxiety level, which was established by comparing the anxiety levels before and after the treatment. The STAI is a popular method of evaluating anxiety. It has 20 items for state anxiety, each graded on a 4-point scale ranging from "1 = almost never" to "4 = very usually." Higher scores indicate higher anxiety levels; mild anxiety runs from 20 to 39, moderate anxiety ranges from 40 to 59, and increased anxiety ranges from 60 to 80 [6]. Internal consistency coefficients for the STAI have varied between 0.86 and 0.95, and test–retest composite reliability has varied between 0.65 and 0.75. [21] Additionally widely utilized in Chinese populations, [22] STAI has been used in numerous investigations. At each time point, vital signs (RR, HR, DBP, and SBP) were also recorded.

### Study confounders

The feeling of pain intensity and degree of anxiety are both highly correlated with inadequate sleep [23]. The RCSQ (Richards-Campbell Sleep Questionnaire) was also applied to assess baseline sleep quality [24]. The RCSQ is a six-item visual analogue scale created to measure how severely sick patients perceive their sleep quality. The scale assesses noise, overall quality of sleep, time awake, number of waking, sleep start delay, and perceptions of sleep depth. The scale ranges from 0 to 100, with 100 representing a good night's sleep and 0 representing a bad night for the individual. This sample's validity study revealed a whole-scale Cronbach alpha of 0.90. RCSQ is also used expansively in Chinese populations [24].

### Procedure

Both the experimental and control groups received binaural headphones from the senior, experienced researchers. Three-morning sessions of binaural headphone delivery were administered to the experimental group (2 h after extubation, the second day following transfer to the general ward, and 1 day before discharge). 15 min of music therapy using a binaural headphones was performed with the participants lying in a recumbent position after a 30-min rest. Since it has been discovered that melodious music with calming rhythms can have a calming effect and cause individuals to feel good [25], the same low-key music with approximately 60–80 beats per minute was made available [26]. In addition, before we designed the music genre, we considered that different music styles might have different effects on pain and anxiety of patients after cardiac surgery [27]. Therefore, to avoid confusion of different music types, the same type of music was selected to be transferred to the MP3 players before the intervention, with binaural headphones connected, and volume was regulated by participants.

The patient and setting were properly set up for the intervention (a performance place where mobile phones were switched off, the door was closed, and distractions were removed). The researchers made an effort to avoid disturbing the participants [28]. The control group was given the same setting and identical pre- and post-testing procedures as the experimental group, except for soft music intervention. The study identified the participants' vital signs before the session after measuring their awareness and emotional condition (noticeable symptoms of delirium, anger, depression, etc., are considered unsuitable for intervention). Pain, anxiety levels and vital signs were recorded four times: before, and ten minutes after three corresponding musical therapy sessions. After all data had been collected, a survey was conducted to collect participants' opinions about the music intervention. Before the intervention, the RCSQ was utilized to obtain a baseline measure of the participants' sleep quality.

### Statistical analysis

The Shapiro–Wilk test was employed for data distribution assessment. Continuous data were provided as means ± standard deviations. The student t-tests were carried out for inter-group studies for normally distributed data. Data with non-normal distribution are provided as medians and percentiles (interquartile range between the 25th and 75th percentiles) and analyzed via the non-parametric Mann–Whitney U test. Repeated measures of analysis of variance (RENOVA) with posthoc tests were employed to analyze pain, anxiety, SBP, DBP, HR, and RR within groups. Measurements from RCSQ were used as a covariate since research has shown that participants' pain intensity and anxiety level are severely impacted by deprived sleep quality during the preoperative and postoperative periods [22]. Both the chi-squared test and Fisher's exact test were used to analyze categorical variables. Statistical significance was defined as a *p*-value < 0.05.

## Results

**Fig. 1:**
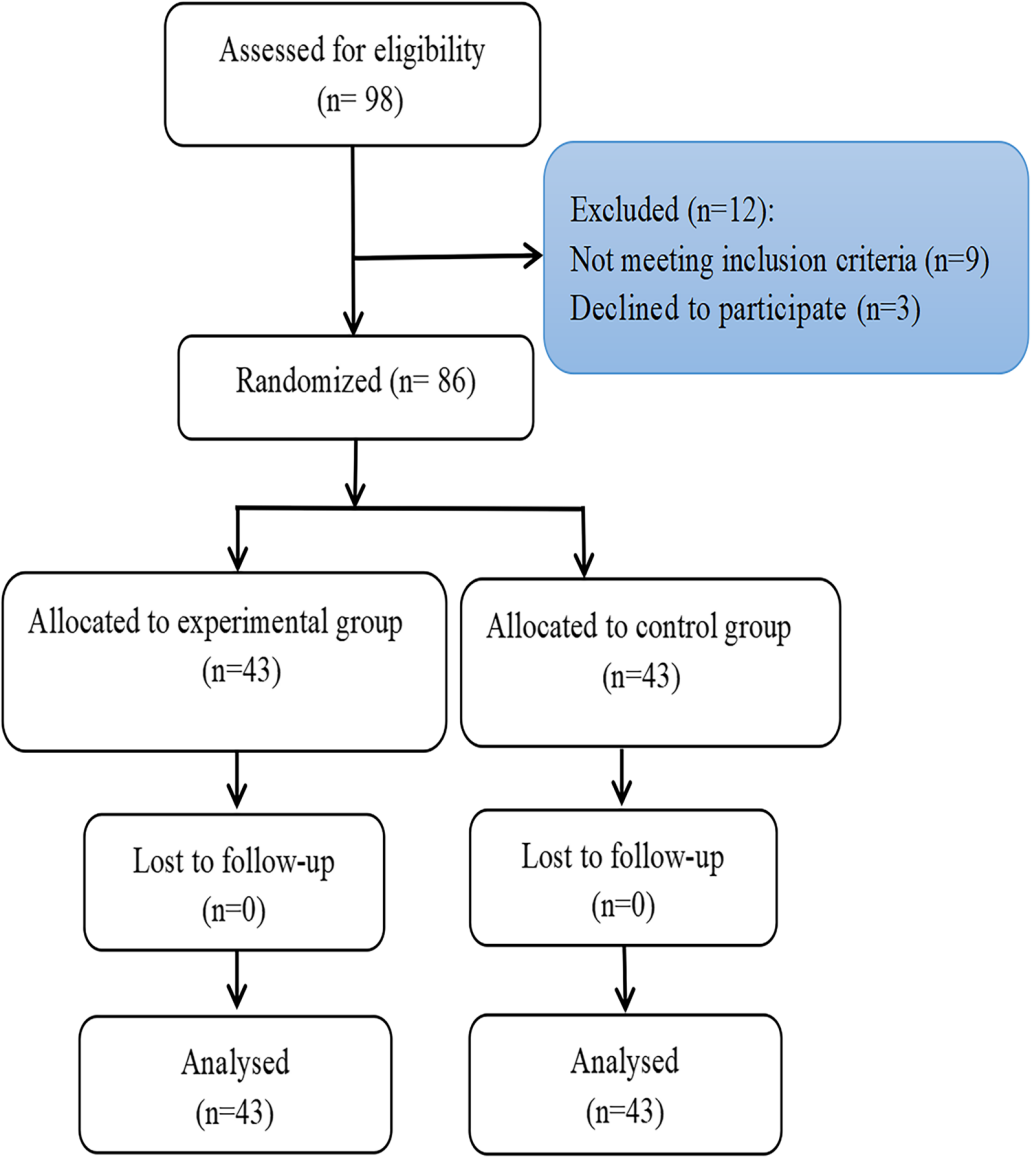
Flowchart showing patient recruitment and randomization

12 of the 98 patients were excluded after evaluating for eligibility. To achieve this, 86 patients were randomly selected and subdivided into 2 groups: 43 underwent the music intervention (experimental group), whereas 43 did not (control group) (see Fig. 1).

### Participants' demographics and baseline features

The individuals' demographic information and basic features are displayed in Tables 1 and 2. Since this investigation was a randomized controlled clinical trial, there were no significant distinctions between the groups in terms of demographic and clinical variables. The two groups' median ages and gender distributions were comparable (*P* = 0.285 and *P* = 0.662, respectively). Between the two groups, there was no difference (*P* = 0.196) in the type of valve replacement ratio. The experimental group's and the control group's respective average intubation times were 11 (9–15) hours and 10 (9–15) hours (*P* = 0.878) and other demographic characteristics see Table 1.

**Table 1:** The clinical characteristics of the two groups

Variables	Experimental group (n = 43)	Control group (n = 43)	t/χ^2^/Z	*P*
Demographics				
Age (years)	57.6 ± 12.7	54.8 ± 11.4	1.076	0.285
Gender, n (%)			0.191	0.662
Male	24(55.8)	26(60.5)		
Female	19(44.2)	17(39.5)		
Education level, n (%)			0.345	0.841
Primary and below	20 (46.5)	22 (51.2)		
Middle school	14 (32.6)	14 (32.6)		
High school and above	9 (20.9)	7 (16.3)		
Nature of occupation, n (%)			0.187	0.665
Manual worker	22 (51.2)	24 (55.8)		
Non-manual worker	21 (48.8)	19 (44.2)		
Marital status, n (%)			0.378	1.000
Single	4 (9.3)	3 (7.0)		
Married	33 (76.7)	34 (79.1)		
Divorced	3 (7.0)	3 (7.0)		
Widow	3 (7.0)	3 (7.0)		
Hospitalization payment, n (%)			0.081	0.776
Own expense	7 (16.3)	8 (18.6)		
Health insurance	36 (83.7)	35 (81.4)		
Religious belief, n (%)			–	0.676
No	39 (90.7)	41 (95.3)		
Yes	4 (9.3)	2 (4.7)		
Previous non-cardiac surgery, n (%)			–	1.000
No	40 (93.0)	41 (95.3)		
Yes	3 (7.0)	2 (4.7)		
LVEF (%), median (Q1;Q3)	55 (50; 65)	59 (52; 64)	2.985	0.412
Underlying conditions				
History of hypertension, n (%)			0.091	0.763
No	36 (83.7)	37 (86.0)		
Yes	7 (16.3)	6 (14.0)		
History of diabetes, n (%)			0.104	0.747
No	36 (86.0)	37 (88.4)		
Yes	6 (14.0)	5 (11.6)		
Abnormal thyroid function, n (%)			–	1.000
No	37 (90.7)	36 (93.0)		
Yes	4 (9.3)	3 (7.0)		
Replacement valve type, n (%)			1.675	0.196
Mechanical valve	19 (44.2)	25 (58.1)		
Biological valve	24 (55.8)	18 (41.9)		
Intubation time, (hours)	11 (9; 15)	10 (9; 15)	1.293	0.878
ICU stay time, (hours)	22 (19; 23)	21 (18; 23)	1.868	0.626

Data are presented as mean ± SD or median (interquartile range), categorical variables are presented as number (%)

*Q1* first quartile, *Q3* third quartile, *LVEF* left ventricular ejection fraction, *ICU* intensive care unit

*Means the proportion of patients was significantly different between the two groups by Bonferroni method

**Table 2:** The baseline assessment of major study variables

Variables	Experimental group (n = 43)	Control group (n = 43)	t	*P*
Pain	7.46 ± 2.16	7.77 ± 2.02	0.687	0.494
Anxiety	55.88 ± 8.40	57.52 ± 7.98	0.928	0.356
Sleep quality^#^	27.78 ± 19.66	30.05 ± 19.98	0.531	0.597
Vital signs				
SBP	130.45 ± 12.36	131.60 ± 14.75	0.392	0.696
DBP	81.98 ± 6.58	82.71 ± 7.54	0.478	0.634
HR	86.82 ± 14.35	87.58 ± 13.93	0.249	0.804
RR	19.53 ± 2.65	20.21 ± 3.07	1.099	0.275

Data are presented as mean ± SD

*SBP* systolic blood pressure, *DBP* diastolic blood pressure, *HR* heart rate, *RR* respiratory rate

^#^Average sleep quality score of preoperative 3 days

There were no detectable variations (all *P* > 0.05) between the groups in terms of baseline pain, anxiety, vital signs (RR, HR, DBP, and SBP), or sleep quality. The mean pain score of the experimental group and control group were similar (7.46 ± 2.16 vs 7.77 ± 2.02, *P* = 0.494) on the 0–10 Visual Analog Scale (VAS), indicating obvious postoperative pain. On the 0–100 state-trait anxiety inventory (STAI), the control and experimental group anxiety scores showed a similar trend (55.88 ± 8.40 vs 57.52 ± 7.98, *P* = 0.356), demonstrating mild postoperative anxiety. According to the Richards Campbell Sleep Questionnaire (RCSQ) (0–100), the experimental group and control group's respective mean sleep quality scores were 27.78 ± 19.66 and 30.05 ± 19.98 (*P* = 0.597), indicating inadequate sleep (Table 2).

### Primary outcome

#### Pain intensity

A statistically significant interaction between groups and time (pretest–posttest) on pain severity was found (Interaction *P* < 0.001; Table 3). As a result, a simple effect for group and time (pretest–posttest) was evaluated.

**Table 3:** Repeated measures ANOVA on major study variables of two groups

Variables	Experimental group (n = 43)	Control group (n = 43)	LS mean (95% CI)	t	*P*	Time*Group
Pain						Interaction *P*
1st Post-test	6.02 ± 2.10	7.01 ± 2.22	1.04 (0.76–1.32)^#^	0.798	0.030^##^	< 0.001*
2nd Post-test	4.40 ± 1.38	5.87 ± 1.50	1.30 (0.87–1.42)^#^	5.021	< 0.001^##^	
3rd Post-test	2.03 ± 1.26	3.85 ± 1.24	1.72 (1.14–2.09)^#^	7.854	< 0.001^##^	
F*	146.36	88.43				
*P*	< 0.001*	< 0.001*				
Anxiety						Interaction *P*
1st Post-test	52.10 ± 5.50	54.79 ± 6.72	2.98 (1.68–3.65)^#^	3.132	0.002^##^	< 0.001*
2nd Post-test	42.73 ± 4.92	46.62 ± 6.51	4.80 (3.96–5.37)^#^	4.138	< 0.001^##^	
3rd Post-test	35.53 ± 5.08	41.43 ± 5.49	7.21 (5.92–9.10)^#^	6.214	< 0.001^##^	
F*	117.44	49.38				
* P*	< 0.001*	< 0.001*				
SBP						Interaction *P*
1st Post-test	125.31 ± 12.28	132.02 ± 14.67	8.76 (5.88–9.58)^#^	3.300	0.001^##^	< 0.001*
2nd Post-test	120.63 ± 10.18	128.68 ± 12.33	9.80 (6.67–11.74)^#^	4.235	< 0.001^##^	
3rd Post-test	114.29 ± 7.67	125.02 ± 8.45	10.51 (8.68–13.25)^#^	6.665	< 0.001^##^	
F*	26.10	1.245				
P	< 0.001*	0.233				
DBP						Interaction *P*
1st Post-test	81.55 ± 7.99	82.77 ± 7.83	1.51 (0.99–2.15)^#^	0.720	0.475^##^	0.242
2nd Post-test	75.86 ± 6.68	77.30 ± 6.60	2.00 (0.99–2.92)^#^	1.103	0.310^##^	
3rd Post-test	71.61 ± 7.51	73.91 ± 6.70	2.22 (1.31–2.54)^#^	1.519	0.137^##^	
F*	1.510	1.427				
P	0.198	0.203				
HR						Interaction *P*
1st Post-test	83.61 ± 10.71	88.62 ± 10.86	8.99 (5.95–11.08)^#^	2.670	0.010^##^	< 0.001*
2nd Post-test	80.63 ± 9.22	86.57 ± 8.89	8.80 (6.87–12.34)^#^	3.301	0.001^##^	
3rd Post-test	76.92 ± 9.31	82.21 ± 8.92	8.95 (6.84–12.67)^#^	3.153	0.007^##^	
F*	55.57	47.65				
* P*	< 0.001*	< 0.001*				
RR						Interaction *P*
1st Post-test	19.49 ± 2.99	20.21 ± 3.22	2.30 (1.25–3.04)^#^	1.375	0.279^##^	< 0.001*
2nd Post-test	17.51 ± 3.18	19.19 ± 2.43	2.60 (1.58–3.37)^#^	2.853	0.005^##^	
3rd Post-test	17.03 ± 2.53	18.62 ± 2.15	2.44 (1.55–3.44)^#^	4.220	< 0.001^##^	
F*	66.66	58.85				
P	< 0.001*	< 0.001*				

Data are presented as mean ± SD or median (interquartile range), categorical variables are presented as number (%)

F*, Greenhouse–Geisser correction (Failure to pass the Mauchly’s test of sphericity)

LS mean, Least mean difference

*REANOVA* repeated measured analysis of variance, *SBP* systolic blood pressure, *DBP* diastolic blood pressure, *HR* heart rate, *RR* respiratory rate

^##^Analysis of covariance (take baseline value as covariate)

Time = pretest versus posttest; Time*Group = Interaction

**Fig. 2:**
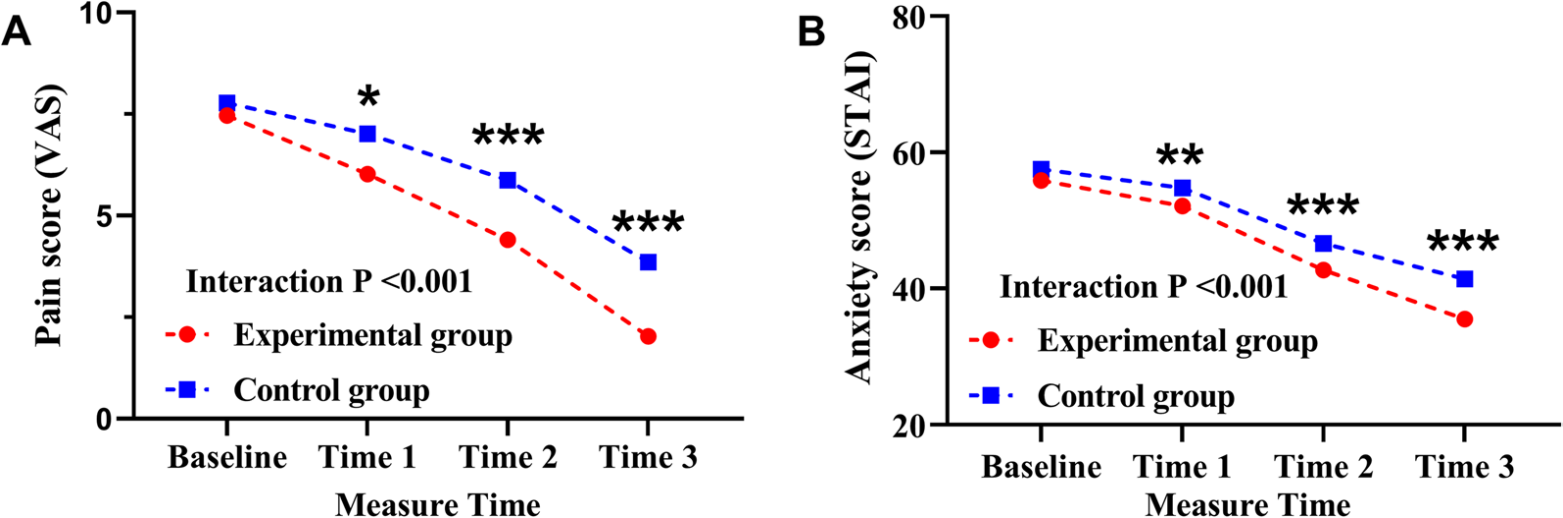
Inter group comparison of Pain score (VAS) and Anxiety score (STAI) at different measure times (mean). Measurements were made at 4 time points: baseline, before music therapy after extubation; Time 1, after music therapy after extubation; Time 2, after music therapy on the second day after transfer to the general ward; and Time 3, after music therapy on the day of 1 day before discharge. (*P < 0.05, **P < 0.01, ***P < 0.001.) VAS, visual analogue scale; STAI, state-trait anxiety inventory. (**A**) Inter group comparison of Pain score (VAS) at different measure times; (**B**) Inter group comparison of Anxiety score (STAI) at different measure times

Using the baseline value as a covariate, the simple effect of group revealed a substantial difference in pain intensity score between the experimental group and the control group: the experimental group reported lower pain compared to control at 1st Post-test (t = 0.798, *p* = 0.030), 2nd Post-test (t = 5.021, *p* =  < 0.001) and at 3rd Post-test (t = 7.854, *p* =  < 0.001), with the Least mean differences (95% CIs) of 1.04 (0.76–1.32), 1.30 (0.87–1.42), 1.72 (1.14–2.09), respectively. According to the simple effect of time (pretest–posttest), there was a significant statistical difference over time in both the control group (F = 88.43, *p* > 0.001) and the experimental group (F = 146.36, *p* < 0.001) (Table 3 and Fig. 2).

### Secondary outcome

#### Anxiety level

A statistically significant interaction between groups and time (pretest–posttest) on anxiety level was found (Interaction *P* < 0.001; Table 3 and Fig. 2). Thus, a simple effect for group and time (pretest–posttest) was evaluated.

The experimental group had lower anxiety level than the control group at the 1st post-test (t = 3.132, *p* = 0.002), 2nd post-test (t = 4.138, *p* =  < 0.001), and 3rd post-test (t = 6.214, *p* =  < 0.001), with Least mean differences (95% CIs) of 2.98 (1.68–3.65), 4.80 (3.96–5.37), and 7.21 (5.92–9.10), respectively. According to the simple effect of time (pretest–posttest), the control group (F = 49.38, *p* < 0.001) and the experimental group (F = 117.44, *p* < 0.001) revealed a statistically substantial decline in post-test anxiety scores compared to the pretest score.

#### Vital signs

There were also significant interactions between groups and time (pretest–posttest) on SBP, HR, and RR (with all Interaction *P* < 0.001), except for DBP (Interaction *P* = 0.242) (Table 3).

**Fig. 3:**
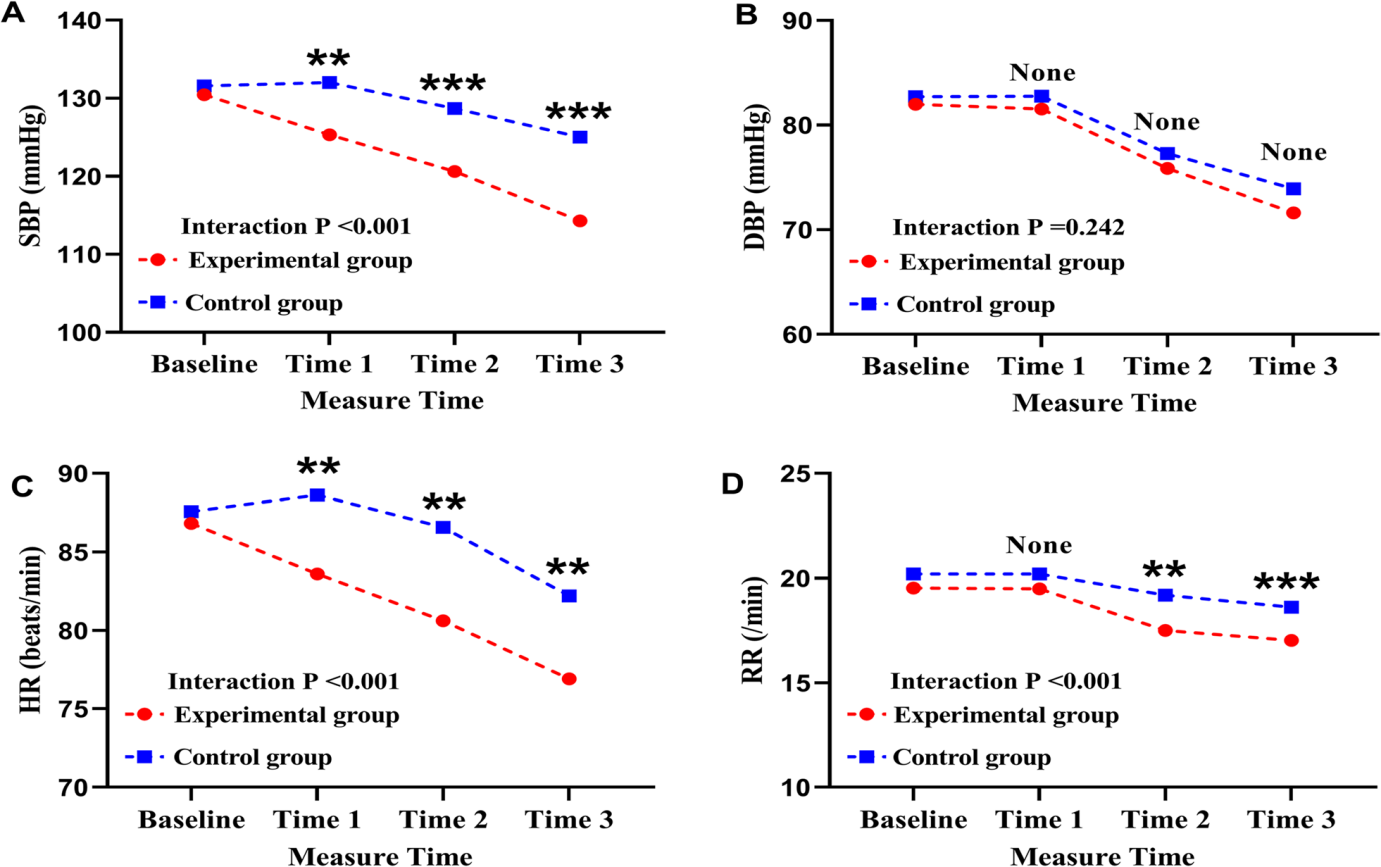
Inter group comparison of vital signs (SBP, DBP, HR, and RR) at different measure times (mean). Measurements were made at 4 time points: baseline, before music therapy after extubation; Time 1, after music therapy after extubation; Time 2, after music therapy on the second day after transfer to the general ward; and Time 3, after music therapy on the day of 1 day before discharge. (*P < 0.05, **P < 0.01, ***P < 0.001.) SBP, systolic blood pressure; DBP, diastolic blood pressure; HR, heart rate; and RR, respiratory rate. (**A**) Inter group comparison of SBP at different measure times; (**B**) Inter group comparison of DBP at different measure times; (**C**) Inter group comparison of HR at different measure times; (**D**) Inter group comparison of RR at different measure times

The simple effect of groups indicated that the experimental group showed reduced SBP at 1st post-test (t = 3.300, *p* = 0.001), 2nd post-test (t = 4.235, *p* =  < 0.001) and at 3rd post-test (t = 6.665, *p* =  < 0.001), with corresponding least mean differences (95% CIs) of 8.76 (5.88–9.58), 9.80 (6.67–11.74), and 10.51 (8.68–13.25); the experimental group had lower HR at 1st Post-test (t = 2.670, *p* = 0.010), 2nd Post-test (t = 3.301, *p* = 0.001) and at 3rd Post-test (t = 3.153, *p* = 0.007), with the Least mean differences (95% CIs) of 8.99 (5.95–11.08), 8.80 (6.87–12.34), 8.95 (6.84–12.67), respectively. Moreover, the experimental group also had lower RR compared to control at 2nd Post-test (t = 2.853, *p* = 0.005) and at 3rd Post-test (t = 4.220, *p* < 0.001), with the Least mean differences (95% CIs) of 2.60 (1.58–3.37), 2.44 (1.55–3.44), respectively (Fig. 3).

The simple effects of time indicated a statistically significant decreased SBP in the experimental group (F = 26.10, *p* < 0.001), HR in the experimental group (F = 55.57, *p* < 0.001) and the control group (F = 47.65, *p* < 0.001), RR in the experimental group (F = 66.66, *p* < 0.001) and the control group (F = 58.85, *p* < 0.001) compared to pretest score (Fig. 3).

### Customer's opinion

97.7% (n = 42) of participants enjoy this type of music, 88.4% (n = 38) felt the music relieved their pain, and 90.7% (n = 39) felt the music alleviated their anxiety.

## Discussion

Numerous procedures, including cardiac catheterization, breast biopsies, cystoscopies, endoscopies, ophthalmologic surgery, extracorporeal lithotripsy and so on have already been explored with music therapy [29].

This first investigation uses music therapy to control pain after heart valve replacement surgery. Assessing the patient's viewpoint is the best way to quantify pain because it is a personal perception. Although other scales are available to assess and measure pain, VASs are the most popular and widely utilized [20]. It is thought that music affects how the brain processes pain, especially by reducing the activity of cortical networks involved in focus and emotional reactions to pain [29, 30]. Music diverts from the unpleasant experience, shifting the attention from the unpleasant to the enjoyable [31]. It was now clearer why, in our trial, music therapy administered to patients who had undergone cardiac surgery resulted in a therapeutically considerable decline in VAS score and a substantial statistical change over the reference condition. Whether the intervention was given in the ICU or the regular wards, the outcomes for pain management were the same.

Additionally, listening to music reduces unpleasant emotional states like anxiety and fear, which have a substantial impact on both how much pain is felt and how it is remembered [32]. Consequently, in our study, those who received music therapy also reported less anxiety in post-test results than in the control group. Our results also showed that the greatest degree of anxiety score relief in the music group was 7.21 (5.92–9.10) on the day before discharge (3rd Post-test), while the smallest degree was 2.98 (1.68–3.65) in the ICU (1rd Post-test). These findings corresponded with the direction of the relief of pain intensity, with the most pain relief of 1.72 (1.14–2.09) on the day before discharge (3rd Post-test) and the smallest pain relief of 1.04 (0.76–1.32) in the ICU (1rd Post-test). This might be the case since most ICU patients have poor sleep, and the benefits of music intervention were underestimated. The benefit of music intervention on pain and anxiety related to poor sleep quality can be somewhat compromised even after correcting for the effects of baseline sleep quality by including it as a covariate.^23^

Previous studies have shown that different patients having the same painful experience at a certain time will perceive less pain than those with less anxiety in the painful experience [9]. This may happen by centrally controlling the emotional component of the pain mechanism. At the same time, it reflects the top-down influence of music therapy's central modulation in peripheral pain perception. It pushes the relationship between this intervention and its outcomes beyond pure psychological processes. Therefore, physiologically, we discovered that music therapy was linked to decreased DBP, SBP, HR, and RR. However, the difference in postoperative cardiac function may affect the fluctuation of BP, HR, and RR measurement results. We lessen these effects by using the average of three measurements of these markers, but we still think a larger sample size is necessary to statistically prove this pattern.

In summary, music therapy helped patients having cardiac valve replacements significantly reduce their discomfort, anxiety, and vital indicators like SBP, HR, and RR. Music therapy seems to be a simple, accessible, and secure technique to enhance the treatment of patients who undergo this operation. At the same time, more research with bigger sample sizes and stronger covariate control is required to validate these findings.

### Limitations

The study has several limitations. First, the music was chosen by the researcher, which may have hindered the effect of music. Second, the researcher was not blinded to the intervention during the data collection procedures. Third, the study was conducted in a single hospital with the result may not be applicable to other postoperative patients.

## Conclusions

This research revealed that music therapy could be used as a complementary treatment for pain and anxiety management following cardiac valve replacements and similar surgical techniques, particularly when relatives are not permitted to visit patients in the ICU due to the COVID-19 pandemic.

## Data Availability

The data that support the findings of this study are available from Fujian Cardiac Medical Center but restrictions apply to the availability of these data, which were used under license for the current study, and so are not publicly available. The Data and full trial protocol are however available from Zeng-Rong Luo author upon reasonable request and with permission of Fujian Cardiac Medical Center.

## Abbreviations

BMI: Body mass index
EF: Ejection fraction
DBP: Diastolic blood pressure
SBP: Systolic blood pressure
RR: Respiration rate
HR: Heart rate
VAS: Visual analogue scale
STAI: State-trait anxiety inventory
RCSQ: Richards-Campbell Sleep Questionnaire
RENOVA: Repeated measures of analysis of variance
ICU: Intensive care unit
CI: Confidence interval
Q1: First quartile
Q3: Third quartile
